# Changes in the stability of coal microstructure under the influence of weak electromagnetic fields

**DOI:** 10.1038/s41598-024-51575-w

**Published:** 2024-01-15

**Authors:** Oleg Bazaluk, Valerii Sobolev, Oleksandr Molchanov, Oleksandr Burchak, Kostiantyn Bezruchko, Nataliia Holub, Olha Tereshkova, Viacheslav Kulivar, Eduard Fedorenko, Vasyl Lozynskyi

**Affiliations:** 1 Belt and Road Initiative Centre for Chinese-European Studies (BRICCES), Guangdong University of Petrochemical Technology, Maoming, 525000 China; 2 Department of Construction, Geotechnics and Geomechanics, Dnipro University of Technology, Dnipro, 49005 Ukraine; 3 Branch for Physics of Mining Processes of the M.S. Poliakov Institute of Geotechnical Mechanics of the National Academy of Sciences of Ukraine, Dnipro, 49005 Ukraine; 4 M.S. Poliakov Institute of Geotechnical Mechanics of the National Academy of Sciences of Ukraine, Dnipro, 49005 Ukraine; 5 Department of General and Structural Geology, Dnipro University of Technology, Dnipro, 49005 Ukraine; 6 Department of Mining Engineering and Education, Dnipro University of Technology, Dnipro, 49005 Ukraine

**Keywords:** Geodynamics, Geochemistry, Chemical physics

## Abstract

The article presents experimental results of research concerning the action of weak electric and magnetic fields on physicochemical transformations in samples of hard coal with a previously destabilized microstructure. The actions of electric and magnetic fields are fundamentally different by many parameters. It has been shown that after treatment with a weak electric field, coal posted an electret potential with an anomalously continuous charge relaxation. Compared to untreated coal samples, the rate of methane emission from methane-saturated samples is maximum for long-flame coal and decreases as it approaches anthracite. The electric field stimulates the grinding of microparticles, a decrease in the maximum gas outlet temperature, a decrease in the enthalpy value in the formation of a new phase, and an increase in the chemical activity of treated coal samples. Fundamentally different results were obtained with magnetic stimulation of coals. In X-ray diffractograms of coal powders after magnetic treatment, the values of the maxima of the main peaks are the largest in comparison with the original samples and those treated with an electric field, which is consistent with an increase in the size of microparticles in a magnetic field. There is an increase in the heat of combustion and a decrease (by 5 times) in the loss of coal mass during heating.

## Introduction

In underground coal mining, one of the greatest unsolved problems of mining science is the physics and chemistry of the nature of the occurrence and development of gas-dynamic phenomena, especially in the form of sudden outbursts of coal and gas. Among the main reasons that arouse interest in the nature of sudden outbursts of coal and gas, are mainly those that relate to current issues of work safety and labor protection (the development of effective methods for suppressing hazardous outburst conditions in coal or preventing outbursts), the research of the fundamental properties of coal and physicochemical effects that manifest themselves under the complex influence of external physical factors on the coal–gas system. The relevance of solving the problem of sudden outbursts of coal and gas is due to the need to study the conditions for the formation of abnormally large volumes of additional methane and the mechanisms for the transition of the solid phase of coal into gas.

Research on the influence of mechanical activation on the destabilization of the microstructure of coal is not limited by the work^1^, however, regardless of the number of works with similar contents, the results differ qualitatively little. The experiments mainly use one factor—mechanical, the influence of which does not sufficiently reflect the real conditions and other impacts on the coal bed. Modeling of microstructural transformations should be envisaged as a complex impact involving weak electric or magnetic fields on the metastable microstructure of coal. In Ref.^1^ (as in the great majority of other studies), the organic mass of coals of a series of metamorphisms from geological disturbances, dangerous and non-hazardous on sudden coal and gas outbursts, was researched. In research of the microstructure of coals, methods of infrared analysis, gas chromatography, and EPR spectroscopy were used, the results of which indicated the connection of outburst-prone coals with geological disturbances. The disadvantage is that the results obtained do not allow us to evaluate the physicochemical features in the transformations of the nanostructure of coals. Our first results in the research on the influence of weak electromagnetic fields^2^ turned out to be similar in nature to transformations initiated in the microstructure by a mechanical factor^3^. In addition, in work^2^, the effect of initiating chemical reactions and phase transformations under the influence of a weak electromagnetic field was experimentally established, which was not observed in all experiments on mechanical activation and was not considered in the general outburst scenario^4^. Electrical activation of coal confirmed the productivity of the idea of using weak electromagnetic fields for structural and phase transformations in hard coals^5^. The advantages of the action of weak electric and magnetic fields on the thermodynamically metastable microstructure of a solids are the activation of the release of additional energy for the development of controlled chemical relaxation and stimulation of electrically and magnetically dependent chemical reactions. The relevance of such scientific research is due to the need to create a unified system of physical ideas about the nature of the formation of outburst-active states in a coal rock massif, phase, and structural (coalification) transformations in coal matter.

It is assumed that the outburst-prone state of coal is due to the property of its microstructure, which is in the ability to remain in a state of unstable equilibrium for an extremely long time, exclusively and only under conditions of an equal stress state. Violation of this condition leads to the formation of a microstructure with a reserve of excess additional energy and the formation of a large amount of gas and explosive development of the coal and gas outburst. The cited works^3–5^ discuss the supposed reasons for the formation of outburst-prone coals and the scenario for the outbursts' development. The physical and chemical mechanisms of some key positions in the processes of outburst and the transition of solid components of coal directly into gas are considered. There has been no systematic research in this direction, as well as research about the action of weak electromagnetic fields on the microstructural transformations of coals (and solids in general). Assumptions were made on the basis of an array of available data from statistical analysis of observations of coal and gas outbursts in mines around the world: Kuzbass^6^, Turkey^7^, Australia^8^, China^9^, Ukraine^10^, etc. The study of the influence of external physical factors (natural and technological) on the formation of physicochemical properties and the formation of extreme energy-intensive states in the microstructure of coals, potentially capable of initiating the development of gas-dynamic processes, will significantly expand knowledge about the causes, conditions, and mechanisms of phase transformations in coals.

## Physical factors stimulating gas formation in coals

The main physical factors that can fundamentally change the stability state of the coal microstructure and its phase composition are mechanical, thermal, electric, and magnetic fields and their various combinations. In this work, the purpose was not to conduct experimental studies of the influence of each of the listed factors in a wide range of values of their parameters. Non-energy-intensive and rational parameters of electrical^5,11^ and magnetic intensities used in experimental research have been established: temperature is up to 325 K, pressure is (2–5 10^5^) Pa, the electric field strength is up to 220 V/cm, the magnetic field is up to 4 × 103 A/m. Research of physicochemical transformations in solids under the action of weak electric and magnetic fields has practically not been carried out. Probably the reason is that the energy of electric and magnetic fields is several orders of magnitude less than the energy of the thermal motion of particles, which raises doubts about the noticeable effect of weak fields impact on any physicochemical transformations in the microstructure of a solids^12^. The effect of the electric field was experimentally proven in research on the solid-phase growth of diamond crystals in the area of its thermodynamic metastability^13^, crushing of metal grains^14^, and quantum mechanical models of the stability of the coal nanostructure under the electric field exposure^15^.

It has been established that outburst-active coals are formed under natural conditions corresponding to the range of action of weak electric and magnetic fields^16^, certain temperatures, and pressures^17^, including, in the numerical analysis of the parameters responsible for the manifestation of sudden outbursts^18^. As a result of a comparative analysis of the physicochemical characteristics of coal samples taken from various areas of coal beds and processed coal samples in laboratory conditions, features of structural and phase transformations are determined, similar to coals from outburst-prone areas.

### Mechanical activation

Rocks of the earth's crust (including coal), especially during periods of tectonic activity, are subjected to such complex types of deformations as shear, compression, stretching, bending, and torsion. In solids, the shift under pressure causes, depending on the pressure value, the formation of solid solutions, new compounds, polymerization, abnormally high chemical activity, the formation of an abnormally high value of the internal specific surface due to the grinding to sizes of the order of 1 μm, an intense deformation, accompanied by an explosion-like nature of the destruction of the substance^19,20^. On the example of the decomposition of copper sulphate under pressure, N.S. Yenikolopov and A.A. Zharov found on the surfaces near the destroyed high-pressure chamber spraying with atomic copper. With deformation under pressure, polymerization of solid monomers occurs, and an abnormally high reactivity of solid bodies is noted^21^. The explosive-like nature of the destruction of coal and rocks was registered in^19^. Mechanical activation of coals of different stages of coalification leads to a change in the kinetic characteristics of the original coal samples, the formation of highly active fuel, and, in general, to the efficiency of the energy use of coals^22^.

Increased destabilization of nanostructured components of coal caused by mechanical activation leads to a decrease in the degree of stability of coal as a whole. During compression with a shift in the organic mass of coal, destructive processes associated with thermal excitation, breaking of chemical bonds, and the generation of radicals occur intensively, which, as paramagnetic centers (PMCs), are registered as signals of electronic paramagnetic resonance (EPR). Since the time of adjustment of electron shells (10^–13^–10^–14^ s) is much less than the time of contact of atoms with each other in the process of deformation, destructive processes arise and develop in the organic mass of coal (OMC), translating part of the mass of OMC into gas.

According to the data given in Ref.^23^, the ratio of the volumes of gas released per ton of coal during outburst to natural gas content is in the range from 0.97 to 11.1. Within this range are the values of similar outburst indicators at mines in different regions of the world. This pattern may indicate a single nature of the formation of outburst-active coals and the mechanism for the development of coal and gas outbursts.

The physical and mathematical model of coal and gas outbursts, presented in Ref.^24^, considers a complex of factors affecting coal and responsible for the formation of outbursts, a model that determines the critical condition for the occurrence of outbursts and predicts all observable phenomena that precede outbursts. In Ref.^25^, the authors dispute the reasons for the degradation of bonds, with regard to the characteristics of the stress–strain state. The main attention is paid to the laws of the kinetics and thermodynamics of the influence of adsorbed methane on the strength properties of coal^26^, and the interactions between thermal and mechanical processes occurring in coal are studied^27^.

The first experiments on the effects of pressure with shear on coal and rock samples were carried out by M.P. Volarovich and colleagues^19^, on the mechanochemical activation of coal by T.M. Khrenkova^28^ and others. However, the influence of the effect of mechanical destruction on the chemical properties of coals and the possible generation of gas was not studied in these works. The role of shear deformation^29^ and destructive processes in the microstructure of coal^30^ are considered as the key factor in the general scenario for the formation of coal and gas outbursts.

Hypothesis^4^, developed in^31^ and represented by a physical and mathematical model^32^, proposes a mechanism for the spontaneous transition of a part in the solid phase of coal into mobile components as a result of the release of additional energy stored by the microstructure under the action of pressure with shear deformation. The amount of gas formed from the decay elements of the unstable microstructure of coal depends on the amount of stored energy both in the process of formation of the outburst hazard state and during the entire outburst process, starting from the moment of formation of the secondary filtration network. In Ref.^4^ it is assumed that the coal is crushed to micro- and nanoscale fractions simultaneously with the release of both additional (newly formed) gas and coal previously accumulated in the pores. The possibility of methane formation during the destruction of coal and directly during the outburst process is confirmed in Ref.^23^ based on the results of FTIR and 13C NMR spectroscopy of coal samples taken from calm areas, outburst zones of the bed, and coal after an outburst.

Authors R.L. Muller and V.S. Popov first proposed the idea of the origin of gas as a product of chemical reactions in coals^33,34^, assigning a decisive role in the preparation and initiating of the energy emission of chemical bonds. They suggested that the least stable component of coal, capable of transforming into gas when unloading the bed under small thermal or mechanical influences, is the so-called fringe. This idea was later developed in Ref.^35^. However, from^36^ it follows that the assumption about the possibility of generating methane, carbon dioxide, and other gases from the fringe of coal matter both during the unloading of the coal bed and according to data on the manifestation of sudden outbursts is not confirmed.

Since the fringe consists mainly of carbon and hydrocarbon chains, an important source of carbon may well be graphene, the transformation of which into a linear chain was carried out in field emission microscopes under the action of ponderomotive forces of an electric field^37^. Field-based electron images of the molecular orbitals of monoatomic carbon chains indicate that the induced excess charge, localized at the end of the chain, creates an axial load of (3.5–5.2) × 10^–9^ N, which ensures the sequential breaking of chemical bonds and the pulling of the chain. Breaking of the bonds is accompanied by local heat release, while the amplitude of atomic oscillations corresponds to a temperature of 104 K^38^. A temperature of this order can contribute to the separation of the chain from graphene and its further decay^37^. As the modeling of the dynamics of the chemical act shows^15^, the state of stability of the chain (carbon or hydrocarbon) is determined, in particular, by the number of carbon atoms.

Thus, in connection with the possibility of transformation of graphene into linear chains and their transition to a state of instability under the action of an electric field, high temperature, and possibly shear deformations, it is assumed that graphene can be an additional source of atomic carbon for the formation of methane in coals.

### Thermal and thermobaric activation

In addition to mechanical effects, heat activation is used to destabilize the microstructure of coal^39^. The results of differential thermal analysis and spectroscopic studies of coal gases, obtained by simple heating of samples consisting of a mixture of coals (coking coal + fat coal)^40^ and anthracite^41^, are given in Table 1.

**Table 1:** The temperature of the maximum release of gases from coal under heating.

Gas molecules	A mixture of fat coal and coking coal	Anthracite
N_2_, O_2_, CO	Desorption of gases from the surface layers at 300 K;	–
H_2_O, OH^+^	Maximum release in the range of 320–390 K; second maximum 700 K	Maximum release at 480 K and 710 K;
CO_2_; CO	Intensive release at 600 K; maximum release at 650 K	Intensive release in the range of 450–680 K; maximum release at 720–740 K
CH_4_	Low release (surface evaporation) 300–600 K; intensive release begins at 650 K; the maximum release corresponds to 720 K	Low release (surface evaporation) 300–700 K; intensive release at 720 K; maximum release at 900 K
	Release of aromatic and aliphatic compounds 740 K and more	Release of aromatic and aliphatic compounds 900 K and more

Possible ratios of the current values of the above-listed physical factors, periodically removing the microstructure of coal from a stable equilibrium, thus stimulating the development of coalification, remain unknown. However, a change in the magnitude of any of these factors (or equilibrium conditions) will bring the system out of a state of stable equilibrium, enhancing the physicochemical processes in the system aimed at counteracting changes.

Paper^39^ reports the results of the gasification of coal during its hydrogenation (temperature, up to 850 K; pressure, up to 3 × 10^7^ Pa) and indirect liquefaction (obtaining synthesis gas followed by catalytic synthesis of hydrocarbons). Chemical processes occurring at large values of pressure and temperature (as in hydrogenation) are not considered in this work since such a combination of values of these parameters is not typical for fossil coals in natural occurrence.

The parameters responsible for the evolution of the physicochemical properties of coal at the stages of coalification from long-flame to anthracite inclusive take into account, as a rule, the values of temperature and pressure^42^. However, the results of numerous experiments did not confirm this assumption, that is, it is not possible to obtain synthetic coal. In this regard, experimental evidence of the formation of geomacromolecules from lipids of plant leaves is of interest^43^. However, the effect of a temperature of 623 K and a pressure of P = 7 × 10^7^ Pa on plant tissues does not lead to the formation of a stable non-hydrolyzable aliphatic macromolecule similar to kerogen. The authors of work^44^ believe that "… labile alkyl compounds can be a source of an insoluble aliphatic component of fossil organic matter and kerogen in the absence of a stable aliphatic precursor (e.g., cutin) in a living organism".

Considering the results of the action on the coal of mechanical, thermal thermobaric, and thermomechanical treatments, an answer was not found to the question of the cause that stimulates the formation of electrically conductive coals, that is, reasons have not been established for the transition of coal-dielectric to anthracite (conductor). Without comprehensive physicochemical studies, it is not possible to judge the transition to the next stage of coalification only by the total carbon content in the treated coal samples.

## Influence of geological processes on coal properties

Taking into consideration the thermochemical properties of coals^45^, an analysis of the expected conditions for the formation of active zones and the fundamental properties of coal was carried out. It is assumed that the formation of focal zones, potentially capable of developing gas-dynamic phenomena in the form of coal and gas outbursts, is caused by the influence of physical factors caused by geological conditions and processes. It is likely that these zones were formed at certain stages of coalification and that the degradation of the coal microstructure occurs as a result of significant deviations in external physical parameters that determine the degree of stability of a given thermodynamic system.

Within the coal basins (Donetsk^46^, Kuznetsk^47^) and individual minefields, zoning of gas-dynamic phenomena and their connection with geological disturbances has been established, which is explained by the high-stress state of the rocks in the massif^48^. As shown in Refs.^49,50^, geological disturbances are formed by tectonic movements of the earth’s crust. The most dangerous in terms of gas-dynamic phenomena are the areas confined to the intersections of faults^51^ and zones of uplift of the earth’s crust (shear zones)^52^. Faults are characterized by increased permeability and, therefore, are considered as routes for the penetration of fluids and hydrotherms.

An important regularity of coal fields is their spatial confinement to zones of deep basement faults^51^.

Data obtained on the basis of long-term observations^52^ indicate the genetic connection of shear tectonics and the regular spatial distribution of areas with gas-dynamic effects relative to shear zones. One of the indirect evidence of the influence of the tectonic factor on the manifestation of gas-dynamic phenomena can be an analysis of the depths of their manifestations^53^. Chemical processes during mechanical dispersion lead to a natural change in the properties of coals – X-ray diffraction analysis indicates intense amorphization of coals after fine grinding. Depending on the size class, the properties of dispersed coal differ little from the properties of structurally altered coal matter. The coal chemical indicator (volatile yield) of the same coal sample can fundamentally change and correspond to the subsequent grade of coal in the coalification series.

Taking into account the information obtained from various sources^10,43,54^, coal can be represented as a kind of composite consisting of carbon and hydrocarbon chains, with distributed nanocrystals of lignin, humic acid, graphite microcrystallites, carbon two-dimensional crystalline phase, which is most likely graphene, a highly disordered material – amorphous carbon, the amount of which gradually decreases during the carbonification process. Since the main element of coal is carbon, it can be assumed that it is the properties of carbon phases that will have a fundamental impact on the physicochemical properties of coal. For example, according to the results of 1500 analyses of samples of coal and anthracite from the Donets Basin, the distribution of the percentage of carbon content was built^55^. The border between coals and anthracites is determined by a carbon content of 93.8%. For coals containing carbon from 91% to 93.8%, changes in the frequency of occurrence are characterized by a sharp decrease from 25 to 5%.

The abrupt transition from coals to anthracites is probably associated with an increase in the concentration of electrically conductive newly formed carbon nanoscale phases, their ordering and stabilization of structural elements with a sharp change in the physical and mechanical characteristics of matter, for example, electrical conductivity. As is known, anthracites are characterized by an electron type of conduction. The high electrical conductivity of coal may be due to the formation of carbon phases with an electronic type of conductivity. Coal becomes a conductor if the number of conducting phases in its microstructure exceeds some critical value. For example, the scenario of a jump-like transition of siderite (a dielectric mineral) to a conductor with an electronic type of conductivity is due to the spontaneous generation of carbon nanoscale phases in mixtures of calcite powders with silicon^56^ and in siderite^57^ at a temperature of 660 ± 10 K and simultaneous exposure to an electric field with a strength of *E* = 200 V/cm. When heated without being exposed to an electric field, the effect of a jump-like increase in electrical conductivity in siderite is not manifested^58^.

An important feature of carbon as one of the main elements of coal is its absence in the free atomic state (in the form of carbon dioxide) on planet Earth. With the participation of this element, the largest number of chemical compounds, various carbon phases, and their allotropic forms, which are fundamentally different in their physical, mechanical, and chemical properties, have been found and synthesized in nature^59^. Of increased scientific and practical interest are intermediate allotropic forms with *sp*^*n*^-configuration (1 < *n* < 3, *n* ≠ 3), mixed (*sp* + *sp*^*2*^ + *sp*^*3*^) in the form of amorphous carbon, nanotubes, fullerene, graphene, graphine, graphane, etc. The formation of chemical bonds is possible in a wide range of pressures and temperatures. However, the stimuli for phase transitions and structural transformations in addition to pressure and temperature are weak electric and magnetic fields.

In the process of coalification, when the external values of pressure, temperature, and other parameters changed in the coals, supersaturation or supercooling arose in the system, respectively, which led to a change in the degree of concentration of atomic carbon, according to which conditions were created in the structure of the substance for the spontaneous generation of carbon crystalline phases. The change in the concentration of the substance of the condensed system occurs under the influences of mechanical, chemical, and thermal factors, and electric and magnetic fields, obeying the thermodynamic principle of mobile equilibrium (Le Chatelier's principle). At the same time, the microstructure of coal formed in the earth's crust during the process of coalification reacts to changes in any of the parameters of external influences that determine the equilibrium of the system as a whole.

The overwhelming majority of cited works report the results of studies into the dependence of structural and phase transformations of fossil coals on the influence of various mechanical and thermal factors, including with their simultaneous impact. Almost all research is aimed at finding solutions to the main problem—the formation of methane in coals—associated with technological problems (methane and coal outbursts, safety and labor protection), economic (increasing the cost of coal), environmental (greenhouse effect), scientific (the physics and chemistry of stability of hydrocarbon phases, phase transformations). The variety of physicochemical processes occurring in rocks (including coals) is due to the influence of such factors as mechanical stresses, temperature, weak electric and magnetic fields, the presence of fluids, features of chemical composition, aggregate state, etc. Among those listed, the mechanisms of action on the microstructure of coal matter by electric and magnetic fields of weak intensities have been little studied, and considering the established effects as a result of treatments with an electric field^5^ and a magnetic field^12^, they require further physicochemical experimental research. Analysis of structural and phase transformations established experimentally on various grades of coal, as well as physical interpretation of the results obtained, can only be complete when compared and assessed with similar changes in coals in nature.

## Results and discussion

### Activation of coal by an electric field

The research used methods of processing coal samples with weak electric and magnetic fields^2,12^ and the design of electromagnetic processing devices^56,57,60^. The experiments used gas coal (carbon content, 86.6%; hydrogen, 5.7%; reflectance index of vitrinite *R*_*0*_ ≥ 0.83%, *Y* = 14 mm, *V*^*daf*^ = 36.2%) and fat coal (carbon content, 88.2%; hydrogen, 3.9%; reflectance index of vitrinite *R*_*0*_ ≥ 1.09%, *Y* = 17 mm, *V*^*daf*^ = 31.3%). The effect of the electromagnetic field was carried out on the coal sample, which was at low pressure (up to 5 × 10^5^ Pa) and temperature (up to 320 ± 5 K). The design of the ceramic container with coal excludes the possibility of oxygen from the surrounding atmosphere entering the chemical reaction zone and prevents the leakage of gaseous products from the reaction zone. The original fractional composition of coal microparticles corresponded to the range of 91–214 μm. Electric current passed through a coil of an electric furnace, excites a pulsating magnetic field in the coal samples. The scheme of the device for the electrophysical processing of coal samples is given in Ref.^61^.

Electrical parameters and temperature were recorded continuously throughout the treatment process. X-ray analysis of the samples before and after the treatments was carried out at the DRON 3.0 unit, taking into account the recommendations given in Ref.^62^. The temperature of the sample was monitored continuously using digital voltmeters; control over changes in the current passing through the sample and the magnitude of the voltage was carried out by voltmeters V7-46/1. A number of studies of physical and chemical characteristics were carried out using thermogravimetric analysis and differential scanning calorimetry TGA/DSC Mettler Toledo, optical microscope LEICA DM ILM, laser diffraction particle size analyzer Shimadzu SALD-301V, and calorimeter C-2000 IKA. The infrared spectra of coals were captured on the Nicolet iS10 Fourier IR spectrometer. The use of Fourier IR spectroscopy makes it possible to obtain information about the molecular structure of the carbon substance, analyzing the features of which it is possible to evaluate the properties of the sample and compare the data obtained with the results obtained by other methods. Electron magnetic resonance (EMR) spectra were obtained using the upgraded RE 1301 spectrometer. Studies of spectra obtained by nuclear magnetic resonance (NMR) were carried out according to the procedure from Ref.^63^.

#### The electret state of coals

In fossil coals selected from outburst hazardous zones, an electret state was found, the lifetime of the external electric field of which significantly exceeds Maxwell's relaxation time^64^. The authors established a relationship between the probability of an outburst and the magnitude of the electret charge as a criterion for the tendency of coal to outburst. It is known that samples of coals in an external electric field acquire a microscopic electric moment, becoming electrets^65^. In practice, the excitation of an electret charge in dielectrics is carried out, most often, at temperatures up to 473 K, acting by an external electric field with a strength of 10^3^–10^5^ V/cm^66^. Charging time is up to 180 min.

Comparative analysis of the parameters of the electret potential and the relaxation time of the electret charge was carried out using samples of the original coal taken from the outburst hazardous zones of the coal bed and coal samples from non-hazardous areas treated with a weak electric field. The experiments used the effects of weak electric fields characteristic of areas of the earth's crust during the period of tectonic activity. The conditions under which the samples of crushed coals were processed had the following characteristics: temperature, 315 ± 5 K; external pulsating electric field strength, 200 ± 10 V/cm; frequency, 50 Hz; processing time, 240 min. In our previously published works^5,67^ it was found that increasing the processing time of coal samples to 48 h does not lead to a significant increase in electret charge and its relaxation time. Samples of crushed coals after treatment with an electric field acquired an electret potential difference (induced potential—*U*_*i.p*._), which was determined by a compensation technique^68^; thermal depolarization analysis was also used in the studies^69^.

**Figure 1:**
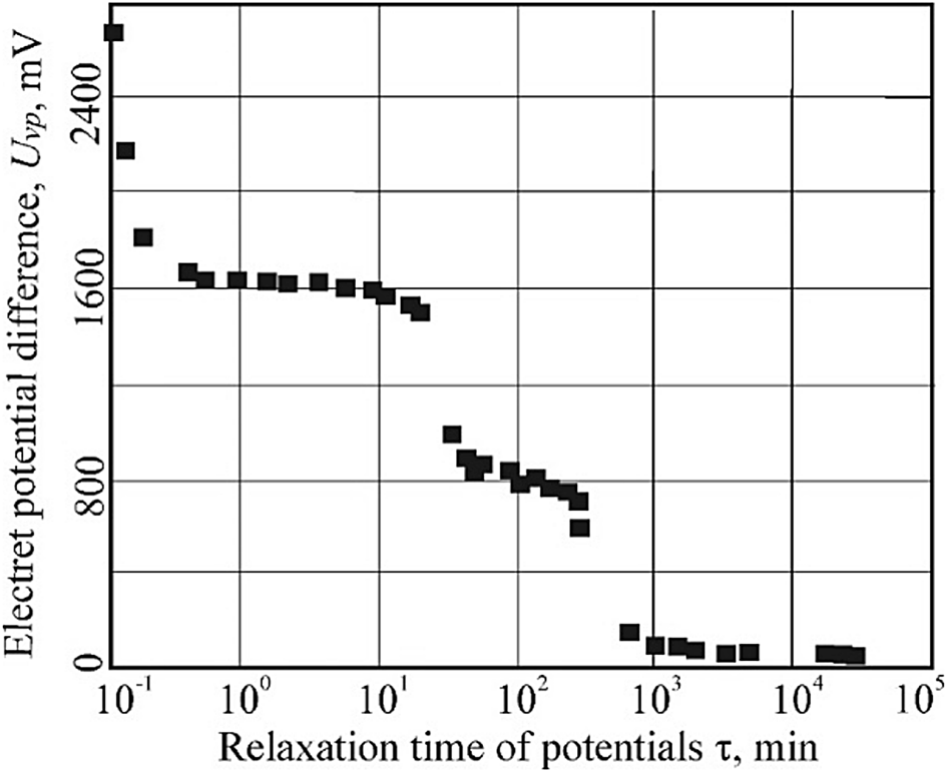
Dependence of relaxation of the electret potential on time in a fat coal sample.

The dependence of change in the electret potential *U*_*i.p*._ on time, Fig. 1, indicates an ultra-slow relaxation of the electret state excited by a weak field.

Analyzing the experimental results obtained on coals not treated and treated with an electric field (non-hazardous, outburst hazardous, after an outburst, mechanized, and after mechanical activation), it was found that the relaxation time of the electret state increases with an increase in the concentration of structural defects in coals. In the microstructure of coal, the energy reserve accumulated in defects of the crystal structure of metals^70^ increases accordingly.

Paper^64^ shows that in the transition zone between "calm" coal and outburst hazardous coal, there is a correlation between the concentration of defects and their energy parameters with the macroscopic properties of coal. In our opinion, in this zone of the coal bed, the concentration of charges increases due to an increase in the concentration of defects, which indicates, in general, an increase in the degree of destabilization in the microstructures of coal.

The nature of the relaxation of the electret charge may be related to such phenomena as the mobility of the excess charge that occurs on the surface of one of the sides of the dielectric during contact electrification^71^, which also depends on the magnitude of the voltage at the ends of the dielectric, the magnitude of the excess charge, the time of charge transfer in the non-conductive dielectric, the interaction of the moving front of the excess charge with the ohmic currents that shield it.

Part of the values of the induced electrochemical activity A_B_ for fat coal, selected from the quiescent zone of the bed, and after a sudden outburst (mine named after A.F. Zasyadko, Ukraine) is taken from work^67^. Here are the values of the stability of the electret state of the induced electrochemical activity and the rate of relaxation of electret potentials in coals treated with an electric field. Measurements of relaxation parameters were carried out at room temperature under conditions of natural humidity. The relaxation parameters of the electret state were recorded until the beginning of the 15th day. Experiments showed that the concentration of defects increases as a result of the mechanical grinding of coal and with additional treatment with an electric field. With an increase in the processing temperature from room to 320 K, the concentration of paramagnetic centers (PMC), relative to the original mechanized coal, increases by 2…5 times. A comparison of the data given in^64,72^ with the results of our experiments shows similar relaxation times of the electret charge. These studies indicate the destructive nature of the effect on coal of even weak electric fields. Probably, similar results can be expected in the case of the influence of electric fields on coal beds in natural conditions. The existence of a correlation between the ability of coal to accumulate electric charges and the tendency to sudden outbursts of coal and gas was reported in work^64^, but at that time there were no confirmatory experimental results.

In the coal samples taken after the outburst, the values of the electret potential are significantly less than before the outburst^64^. In fat coals, the maximum value of the induced potential U_i.p._ corresponds to the outburst hazard state, and the minimum value corresponds to coals from the outburst zone, in which the potential to excite and develop active chemical reactions is almost exhausted. The greatest rate of decrease in electret charge occurs in the first seconds of relaxation.

The physical features of electrical relaxation in high-impedance materials are described, for example, in Ref.^73^. Many of the materials have practical applications^74^. To explain the experimentally observed values of internal electric fields, the presence of polarization in the electret was assumed. A quasi-stationary electric current can exist both in spatial heterogeneity and in the absence of electric fields^75^. In dielectrics that have their polar direction, there is enough disequilibrium for the existence of an electric current but under conditions of thermodynamic equilibrium, the current turns to zero. At the same time, structural adjustments associated with the transition to an equilibrium state involve overcoming significant potential barriers and can therefore occur very slowly. Thus, using the results of experiments, it should be concluded that the long existence of the external electric field of the electret is due not to stability but to the state of its instability. The degree of thermodynamic stability of a microstructure is a function of the density of defects in the crystal structure^76^ and is proportional to the amount of stored energy of a given microstructure. Probably, the creation and maintenance of an external electric field are due to the internal energy stored by the microstructure during the formation of this electret in nature.

The increase in the chemical activity of crushed coals further treated with a weak electric field may be due to the influence of excess charges as catalysts for chemical reactions. It is known^71^ that uncompensated electric charges in the space surrounding the electret create a quasi-static electric field. Using numerical modeling, the influence of the point charge field on the degree of stability of the chemical bond has been established (using the example of small molecules). Thus, the convergence of the bond with the electric charge at some critical distance leads to a break in the connection^77^. As a result of the treatment of gas and fat coals with a weak electric field, the belonging of these coals to electrets was confirmed.

#### NMR studies of sorption properties of coals

The ^1^H NMR spectra of coal were recorded using the NMR radio spectrometer of wide lines designed by the Institute for Physics of Mining Processes of NAS of Ukraine. The field strength of the permanent magnet is 4600 E, the homogeneity is 2 × 10^–6^ E/cm, and the resonance frequency is 19.6 MHz. Since the spectra obtained by the ^1^H NMR method of wide lines represent the sum of the derivatives from the absorption lines, then for interpolation of the experimental spectra of ^1^H NMR one uses the superposition of the first derivatives of the Lorentz and Gaussian constituent lines^78^.

**Figure 2:**
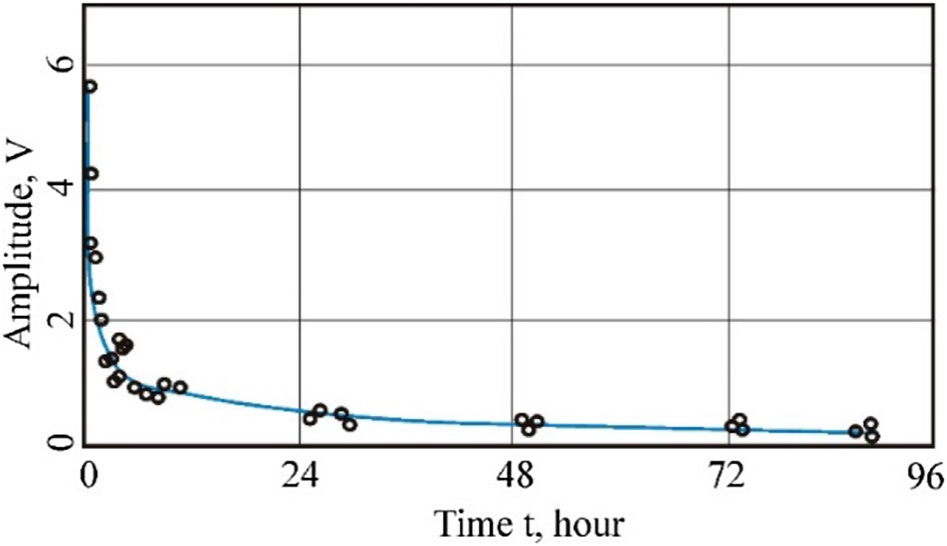
Amplitude of the narrow line of 1H NMR spectrum during the methane desorption from unshredded (whole sample) gas-saturated long-flame coal (grade D).

Samples of long-flame coal taken from the *m*_*3*_ bed of the “Trudovskaya” mine were examined, and the yield of volatile substances *V*^*daf*^ is 42.8%. Whole coal samples were used. Among the coals of a series of coalification, including anthracite, the shortest methane desorption time is in long-flame coal. The dependence of the amplitude of the narrow line of the NMR spectrum of gas-saturated coal (proportional to the number of methane molecules) on time, characterizing the kinetics of methane yield from the sample of long-flame coal, is shown in Fig. 2.

To describe the process of methane desorption from coal samples, as earlier in Ref.^67^, a diffusion-filtration model was followed. At the beginning of desorption, free methane emerges from the sample (from open pores and cracks connected to the outer surface of the sample). Methane, which entered during saturation under pressure into closed pores into macromolecules of a carbon substance like a solid solution, can enter open pores and cracks only by diffusion, and then along the open pores and cracks, it is already filtered outward from coal particles.

Thus, when processing experimental data, we used the approximation dependence in the form of the sum of two exponents:

1 $$A\left({\text{t}}\right)= {A}_{1}\cdot {\text{exp}}\left(-\frac{{\text{x}}}{{t}_{1}}\right)+ {A}_{1}\cdot {\text{exp}}\left(-\frac{{\text{x}}}{{t}_{2}}\right)+ {y}_{0}$$

where *t*_1_ and *t*_2_ are the characteristic desorption times for the two mechanisms; parameters *A*_1_ and *A*_2_ are related to the number of methane molecules in the coal, which is desorbed by the two mechanisms (this is reflected on the spectrum by the amplitude of the NMR signal); *y*_0_ is the shift of dependence along the ordinate axis.

The characteristic times of methane desorption from coal change in the process of coalification, which is associated with transformations of the molecular structure of the coal substance and the fractured-porous structure. Samples of long-flame coal absorb the most methane by the same mass. Compared to other grades of coal, the structure of D-grade coal loses methane faster. The characteristic methane desorption times obtained as a result of experimental data processing for the studied whole sample are equal, respectively, for the diffusion (*t*_1_) mechanism, to 17.4 h, and for the filtration mechanism (*t*_2_), to 0.87 h.

According to the diffusion mechanism, the characteristic time of methane desorption from whole coal samples from long-flame to anthracite increases from two tens of hours to several days and strongly depends on the degree of perfection of the microstructure. It should be expected that mechanical grinding of coal, electrical activation, and other types of influences will lead to a significant reduction in methane desorption time. To test this assumption, gas coal, fat coal, and coking coal were selected, crushed, and a 200/100 μm fraction was isolated for experiments. Half of the sample of each selected grade of coal was treated additionally with an electric field of low intensity (210 V/cm).

The coal samples were dehydrated and then saturated with methane. Studies of the samples were carried out in the contour of the spectrometer, not isolated from atmospheric air. In this regard, in the process of methane saturation, sorption of atmospheric moisture by coal samples simultaneously occurred, the hydrogen of which contributed to the recorded ^1^H NMR spectra. Therefore, on experimental dependences reflecting the change in the amplitude of the narrow NMR line of methane-saturated coal on time during desorption, its gradual drop to zero is not observed. To isolate from the data obtained a dependence reflecting the methane desorption from the sample, it is possible to use the technique described in Ref.^79^. When processing the results obtained, an interpolation dependence of the following form was used:

2 $$y=a\cdot {\text{exp}}\left(-\frac{t}{{T}_{des}}+c\left[1-exp\left(\frac{-T}{{T}_{sorp}}\right)\right]\right)$$

where *a* and *c* are the amplitude coefficients related to the amount of methane and water in the sample, *T*_des_ is the characteristic time of methane desorption from the coal sample, *T*_sorp_ is the characteristic time of sorption of atmospheric moisture by the coal sample, *t* is the time of the experiment. The first term in the right-hand side of this expression describes the methane desorption, and the second—is the sorption of atmospheric moisture.

The change in the amplitude of the narrow NMR lines corresponds to the change in the methane content over time, based on which the characteristic time of desorption *T*_*des*_ (Table 2) is determined. The conditions for recording spectra were maintained to be the same.

**Table 2:** Change in methane desorption time *T*_*des*_ from coals, original and treated with an electric field.

Name of coal grade	Methane desorption time from coal, *T*_*des*_, minutes	
	Original (crushed)	Treated (by an electric field)
Gas coal («G»)	25.9	8.6
Fat coal («F»)	39.1	21.8
Coke coal («C»)	47.7	38.1

**Figure 3:**
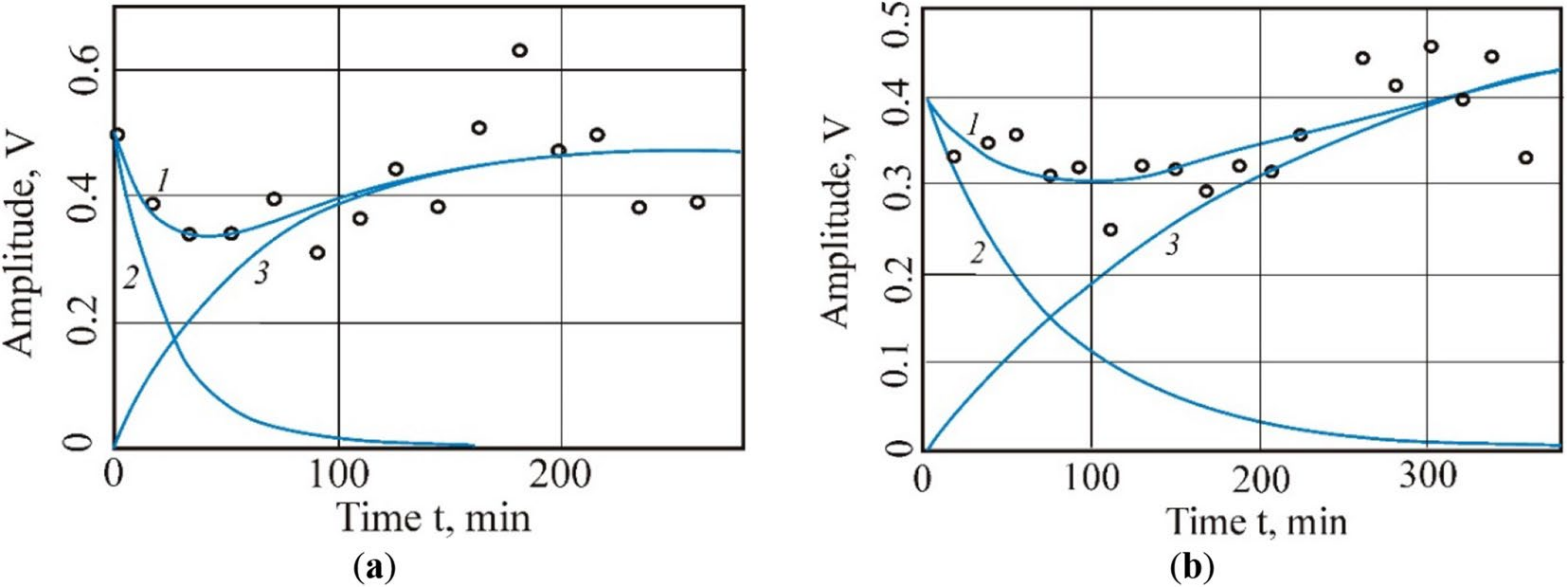
Amplitude of the narrow line of the 1H NMR spectrum during the methane desorption from gas coal: (**a**) original gas coal; (**b**) treated gas coal.

Paper^67^ shows the graphical dependences of change in the amplitude of the narrow line of the ^1^H NMR spectrum of methane-saturated coal samples during methane desorption; it gives the decomposition of the resulting dependence (1) into curves 2 and 3. Figure 3 shows an example of the dependence of the amplitude change during the desorption of methane from gas coal. The points on the charts indicate the sorption of atmospheric moisture by coal samples. In the original methane-saturated coal samples, the methane desorption time increases with an increasing degree of coalification and is reduced for coals treated with an electric field.

A feature of the results of the study of the spectra of gas and fat coals after treatment with an electric field, obtained by the ^1^H NMR method, is a significant decrease in the characteristic time of methane desorption from the treated coals. Desorption from methane-saturated coal treated with an electric field, in comparison with the original coal, decreases by only 3–5%, but at the same time, the rate of methane release increases significantly. So, for gas coal, the release rate of methane is three times higher, for fat coal—1.7 times, for coking—1.24 times; Table 2. The decrease in gas saturation of the treated coal samples is due to the additional release of methane by the organic mass of coal, in which the destructurization processes underwent exposure to a weak electric field.

It has been experimentally shown that as a result of external physical and mechanical influences in coals, destabilizing processes take place, the density of defects, as well as the specific surface area of the coal substance, increase, which as a result leads to anomalous desorption of methane in comparison with solid coal samples. The effect is especially strong with mechanoelectric activation. Perhaps such effects in natural conditions are one of the factors involved in initiating the destructurization of coals and their transition to an outburst hazardous state.

A similar trend in the nature of the change in methane desorption time was noted in the study of coal samples taken from beds in hazardous areas. Thus, paper^78^ reports the results of methane desorption from coal in calm zones and the zone of manifestation of sudden outbursts. The rate of methane desorption differed slightly obviously because samples were taken from a bed with a disturbed microstructure. Coal after outburst is characterized by a low intensity of gas release, which may be due to the loss of reservoir properties. Perhaps the reason for the sharp attenuation of gas release is a decrease in the chemical activity of the organic mass of coal and, as a consequence, a decrease in the reserve of excess energy in the stabilized microstructure.

Studies of mass loss of gas-saturated coal samples due to methane desorption were conducted at a temperature of 363 K using the gravimetric method. The original sample of gas-saturated coal after a time of 100 s loses 0.063 g, after 400 s the mass loss is 0.088 g. The mass loss of the coal treated with an electric field is 0.065 g and 0.075 g, respectively, per 100 and 400 s. A similar pattern is characteristic of gas and coking coal. As the degree of carbonification increases, the mass loss decreases. For the studied coals, the rate of mass loss in the original sample is higher than that in the treated one. As the degree of carbonification increases, coal samples, both initial and electric field treated, are characterized by a decrease in mass loss.

The action of a weak-intensity electric field stimulates the formation of a gas, similar to thermal or mechanochemical activation, and at the same time reduces the temperature of the intense release of gases. Gravimetric studies found that the electret effect in coals is manifested when a weak electric current with a density of 10^–6^–10^–5^ A/mm^2^ and a coal heating temperature not exceeding 320 K. The experiment was terminated after 14 days (20,160 min) with a residual electret potential of 0.6 mV.

The results of the experiments conducted to study the release of gases from electrostimulated coals and the experimental data from the dissertation of S. Lizun (1983) are given in Table 3.

**Table 3:** Mobile components released from fat coal.

No	Type and parameters of coal treatment	Destruction products	Note, literary sources
1	Electroactivation of whole samples T ≤ 340 K, U = 200 V, f ≥ 800 Hz	Light hydrocarbons (LHC); high molecular weight hydrocarbons (HMHC); Gases – O_2_, H_2_O, Ar	Low-intensity peaks LHC and HMHC; Ar content is the highest (S. Lizun)
2	Electroactivation of whole samples T to 320 K, E = to 210 V/cm, f = 50 Hz	Gases – CO_2_, N_2_, CH_4_, O_2_, H_2_O	The composition of components No. 1 and No. 2 are close
3	Electro- and mechanoactivation T = 315 ± 5 K, E = 100–210 V/cm, f = 50 Hz	Radicals – COOH, H, CH Gases – CO_2_, H_2_, CH_4_, Ar, O_2_, H_2_O, C_2_H_6_, C_3_H_8_ etc	The amount of methane almost doubled compared to No. 1 and No. 2

#### Studies of destructive processes in coals

**Figure 4:**
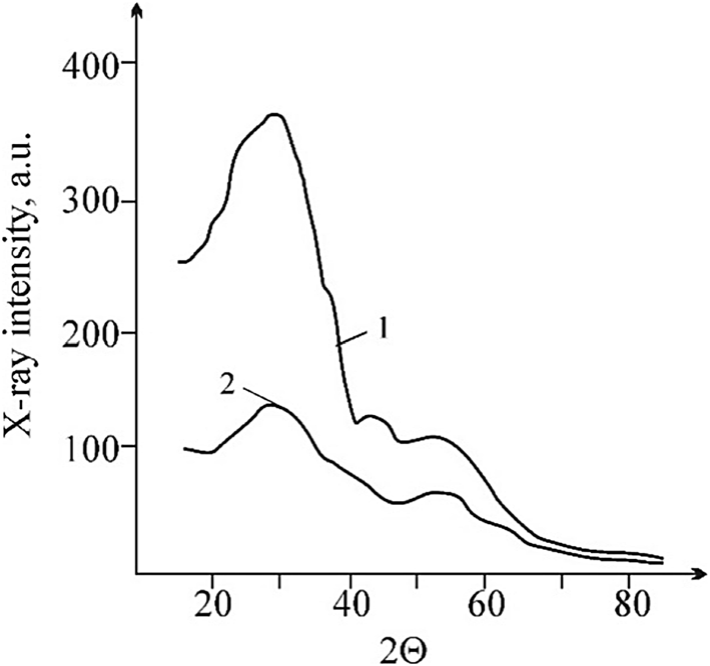
X-ray diffraction pattern of gas coal: 1—the original sample; 2—the sample treated with an electric field.

On the X-ray diffractogram of gas coal, two strongly eroded maxima are distinguished, which correspond to the angles of 2θ: 24 and 43 degrees; Fig. 4^2^. In the region of the first maximum, there are lines whose values are equal (in nm) to: 0.455; 0.424; 0.403—weak line; 0.371 and 0.338. In the region of the second maximum, lines are fixed on the diffractograms of all original samples that can be attributed to the crystalline phase having an interplane distance d = 0.198…0.200 nm, close in value to the second most intense graphite line d = 0.202 nm.

**Figure 5:**
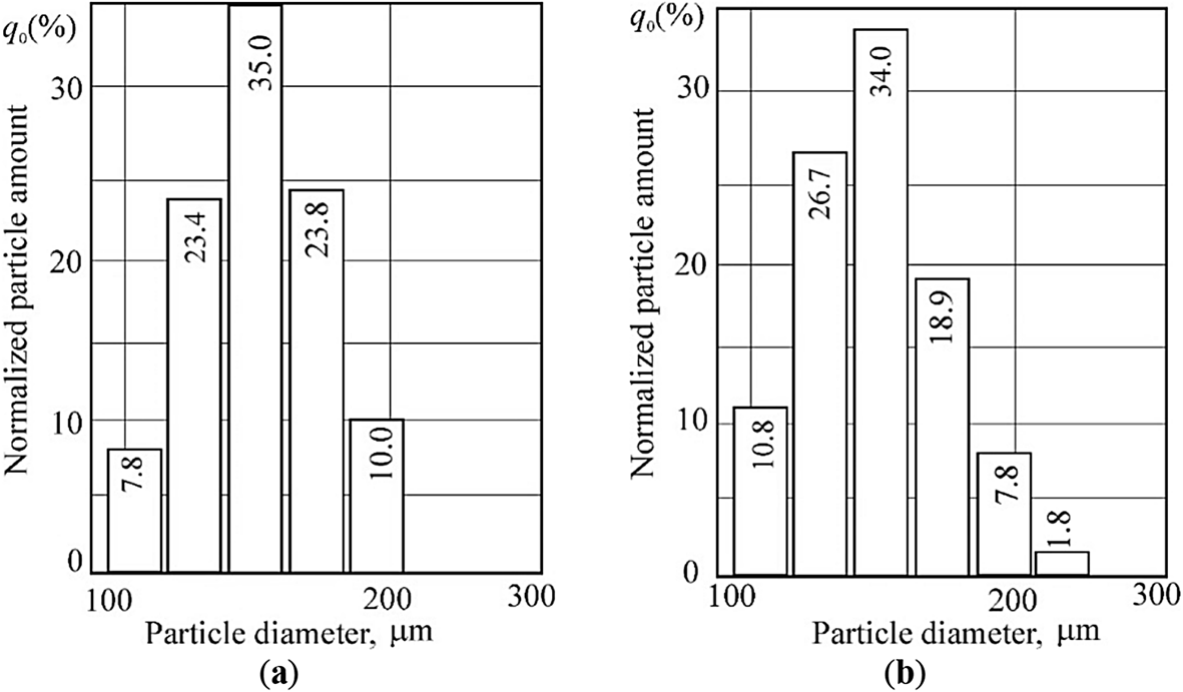
The nature of the distribution of coal particles by size (214–96 μm): 1—the original sample; 2—the sample after treatment with an electric field.

The position of the first maximum varies slightly from sample to sample; the half-width changes slightly. After electrical treatment, the intensity of the coal lines decreased. An increase in half-width indicates, in particular, a decrease in the dispersion of particles, and in general—an increase in the specific internal (sorption) surface and in the degree of "amorphousness"^80^. Destructive processes occurring in coals are accompanied by an increase in the content of smaller fractions in accordance with an increase in the internal specific surface. At the same time, the average particle size and their content decrease by 3–8% Fig. 5.

An increase in the degree of “amorphism” of the structure confirms the nature of the particle size distribution and the increase in the concentration of paramagnetic centers. Coal from the outburst zone has a significantly larger sorption surface than coal from the same bed but is not involved in the outburst^81^. In addition, the dependence of the specific surface of coal on temperature is shown. In our experiments, when treating coal samples with an electric field, in all experiments the range of coal granularity shifted towards smaller particles (Fig. 5).

The development of destructive processes is also evidenced by the results of infrared spectroscopy and electron paramagnetic resonance data^2,82^. In turn, destructurization precedes the transformation of part of the organic mass of coal into the gas phase. For example, the destruction of bridged aliphatic chains is confirmed by a decrease in the optical density of bands of 2920 and 2860 cm^–1^, corresponding to the valence and deformation oscillations of the C–H bonds. These bonds belong to structures that contain CH_2_ and CH_3_-groups and can be caused by a decrease in bands of 3000…3100 cm^–1^ in aromatic hydrocarbons. IR spectra of coal treated in an electric field are similar in nature to the established patterns in the study of coals selected from various areas of outburst-hazardous and non-hazardous coal beds^83^.

According to EPR, the concentration of paramagnetic centers (*N*) in fat coals after grinding increased from *N* = 5.6 × 10^18^ PMC/g to 8.2 × 10^18^ PMC/g. Additional treatment with an electric field leads to an increase in the concentration of PMC: (*N* = 3.3 × 10^19^ PMC/g). In the organic mass of coal of outburst hazardous beds and outburst hazardous areas, for example, according to^29,78^, changes in molecular organization and changes in the structural and chemical transformations occur reflecting the state of mechanical activation of the organic mass of coal. From the analysis of coal EPR lines from outburst hazardous and non-hazardous zones, it follows that the widening of the EPR line (for example, up to 35 mT) for outburst hazardous coal is due to free radicals and is described by the Lorentz equation. The widening of the EPR line occurs in the cases of mechanodestruction, thermal destruction, and electrodestruction of coal, that is, it is mainly due to the thermal mechanism. The rupture of chemical bonds may be due to the high content of impurities in the composition of coals. This trend persists in the case of coal processing in weak magnetic fields. However, in the latter case, the widening of the EPR signal may be associated with the spin exchange in the presence of oxygen and paramagnetic metal ions. Here, the mechanism can be determined by the regularities of the magnetic scenario of chemical reactions^84^.

To obtain additional information on changes in the structure of coal, the studies used nuclear magnetic resonance spectroscopy on carbon-13 nuclei (^13^C NMR) and Raman scattering spectroscopy (CS spectroscopy). Coal was selected from the outburst hazardous zones and at the site after the outburst (coal from the *h*_5_ bed («Krasnolimanskaya» mine, Pokrovsk, Ukraine)). The relationship of coal outburst hazards with the total content of CH_2_- and CH_3_-groups, as well as the ratio of carbon atoms with different hybridization of valence electrons (sp^2^/sp^3^)^85^ was established.

Table 4 gives the CP/MAS ^13^C NMR spectra, acquired by using the cross-polarization (CP) method to detect fragments containing hydrogen in the coal matrix. Thus, in coal samples from outburst hazardous zones (experiment No. 2), an excessive content of CH_3_-groups was found relative to the content of these groups in coals selected from calm zones (experiment No. 1). At the same time, the spectra of outburst hazardous coal also show an increase in the carbon content in sp^2^-state, which is possible with an increase in the content of chain fragments containing CH-groups.

**Table 4:** CP/MAS 13C NMR spectra of coal samples.

Experiment No	Coal samples	Relative intensity of maximum values of spectra, mm			
		CH	CH_2_	CH_3_	CH_3_/CH_2_
1	non-hazardous (calm)	80	33	26	0.79
2	outburst hazardous	138	57	58	1.017
3	after outburst	131	48	46	0.958

The ^13^C NMR spectra of outburst-hazardous coal contain an excessive number of –CH_3_ groups (experiment No. 2) compared to the content of these groups in coals taken from calm zones. In the outburst coal (experiment No. 3), there was a decrease in the -CH_3_ groups to a level corresponding to the calm zone and even less than this level. A decrease in the intensity of the peak from the CH_3_ groups is visible on the spectrum of coal from the outburst zone. In coals from the outburst zone, the ratio of the values of the integral intensities of the CH_3_ and CH_2_ lines (methyl and methylene groups) decreased by 1.5 times. Similar results were obtained for original gas coal (outburst non-hazardous) and that treated with a weak electric field.

The method of cross-polarization (CP/MAS) was used to amplify the signal from carbon atoms by attracting hydrogen atoms in the composition of fragments with ≡C–H-bonds. The implementation of modern possibilities of ^13^C NMR spectroscopy in the study of structural and functional transformations in multimers makes it possible to register not only qualitative changes in materials containing carbon under the influence of various external factors but also to quantify the distribution of carbon atoms between functional groups.

**Figure 6:**
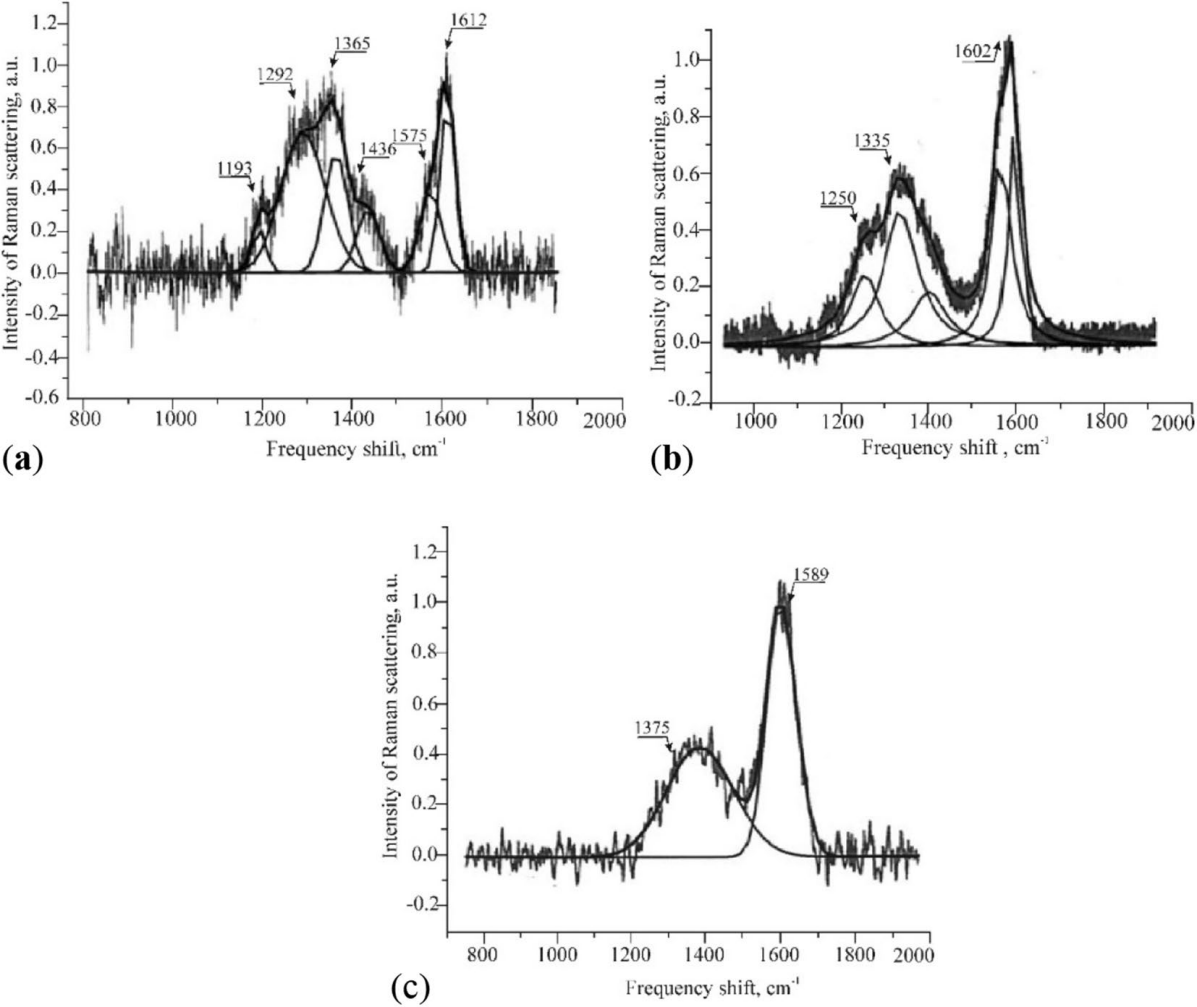
Combined scattering spectra of fat coal (II) from «Skochinsky» mine: (**a**) from the outburst zone; (**b**) from a safe zone; (**c**) after the outburst.

To confirm the NMR spectroscopy data, additional studies were carried out using Raman scattering spectroscopy^86^. Selected samples of coal from bed h_5_ were examined using a Eurolase solid-state laser: wavelength, 0.473 μm; output radiation power, 17 mW. Each spectrum was recorded within 1 min. Processing of the data obtained showed that on the RS spectra of coal samples from outburst zones, Fig. 6a, (compared to samples from calm areas, Fig. 6b), additional bands appeared in the D-band region with a frequency shift value of ~ 1190 cm^−1^ and 1430 cm^−1^. The band of the CS spectrum of the outburst hazardous coal in the frequency shift region of 1160–1190 cm^−1^ is associated with oscillations of C–C bonds in = C–C = groups. The band with a frequency shift value of 1436 cm^−1^ is caused by the appearance of ≡C–C≡ bonds (polyynic chains).

The CS spectra of the outburst-hazardous coal decompose into 6 components (Fig. 6a), while the CS spectra of coal of calm areas (Fig. 6b) not only of the bed* h*_5_, but also of the coals of the coalification series to anthracite maximally, decompose into only 5 components. After the outburst, the CS spectra contain 2 components (Fig. 6c). As a result of the influence of an electric field on the crushed sample of coal taken from the calm zone of the bed *h*_5_, the Raman spectrum of scattering has no fundamental differences from the spectrum of outburst-hazardous coal (Fig. 6a).

The maximum of the G-band shifts from the value of the frequency shift of 1612 cm^−1^ to 1589 cm^−1^, which is associated with the destruction of chain fragments containing CH groups. For the CS-spectrum of the sample of the calm region, the value of the frequency shift of the G-band is 1602 cm^−1^. The change in the nature of the CS spectra of the outburst-hazardous and ejected coal confirms the above conclusion about the increase in the groups of –CH_3_ in outburst hazardous areas and on the further destruction of the organic mass of coal during an outburst by reducing = CH–CH = and –CH_3_ groups. The stability of carbon chains, as shown by the results of numerical simulations^80^, is due to the action of excessive external electric charges and the number of carbon atoms in the chain. Summarizing these facts, we can interpret them as the result of the action on the coal bed of mechanical activation and electric field strength.

The established regularities give grounds to confirm the assumption^87^ that during the outburst methane formation occurs mainly due to the participation of hydrogen, which is included in the groups of =CH_2_ (sp^2^-hybridization) and groups of –CH_3_ (sp^3^-hybridization). The prerequisite for intensive methane formation is the achievement of a critical concentration of –CH_3_ groups and the ratio of hydrogen atoms in the composition of =CH and –CH_3_ groups equal to 4:1.

### Treatment of coal with a weak magnetic field

**Figure 7:**
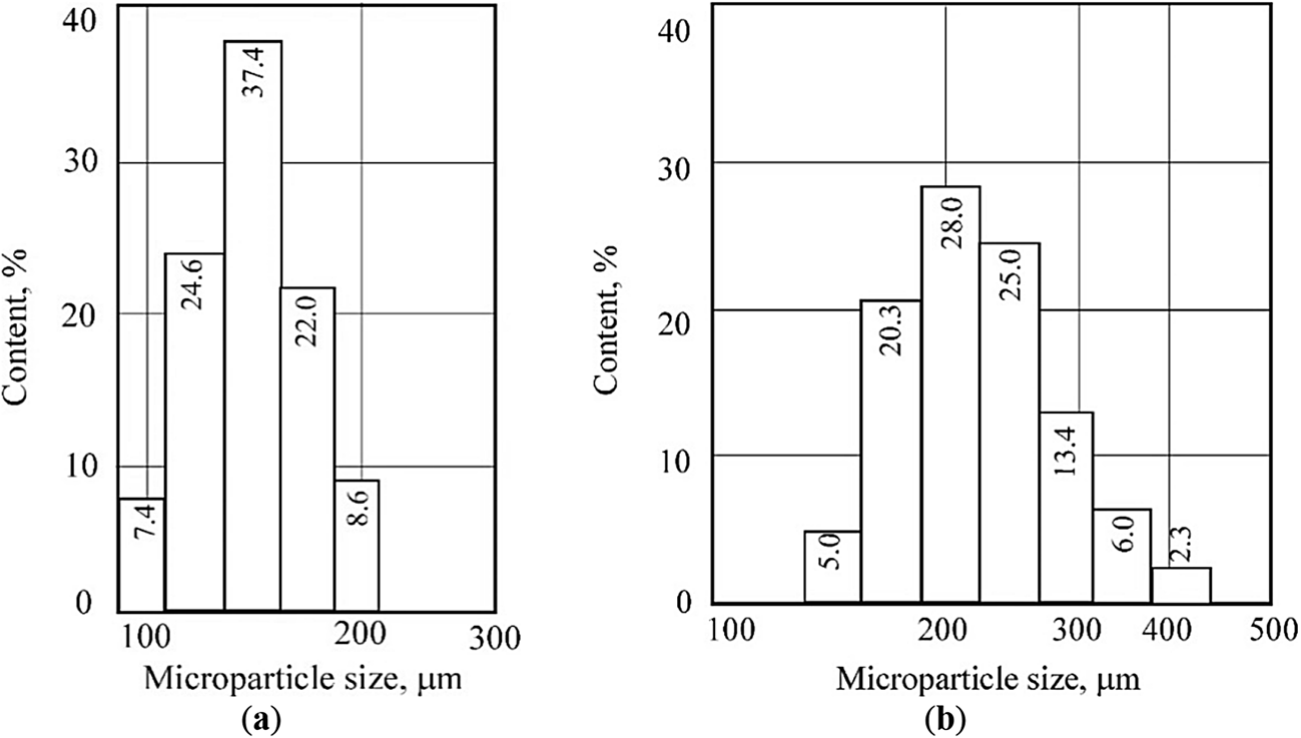
The nature of the distribution of coal particles by size: (**a**) the original sample; (**b**) after treatment with a magnetic field.

One of the main new results of the action of a weak pulsating magnetic field on microparticles of crushed coal (200/100 μm) is an increase in the size of microparticles within the entire range of fractions (Fig. 7). We explain this effect by singlet–triplet transitions, similar to those discovered in Ref.^76^, but only in liquids and gases.

To assess the effect of weak pulses of the magnetic field on the structural characteristics associated with the sorption properties of the carbon substance, modern structural research methods were used—electron paramagnetic resonance (EPR) and infrared spectroscopy (IRS). EPR spectroscopy was used to determine the features of the electronic structure of coal macromolecules, to assess the kinetics of the interaction in the coal–gas system and the threshold sorption capacity of the carbon substance^88^. The results of the assessment of the structural characteristics of coals before and after treatment with a weak magnetic field are given in Table 5. Figure 7 illustrates the distribution of coal particles by size, depicting two distinct scenarios: the original, untreated coal sample and the distribution of coal particles after undergoing a magnetic field treatment.

**Table 5:** Structural characteristics of fat coal obtained by the EPR method.

Size class, μm	Structural characteristics of coal substance			
	*N*	Δ*H*	*K*_*sc*_	*Q*
160–100 (enriched with vitrinite)				
Initial state	2.11	5.57	0.24	5.0
After exposure to a magnetic field	2.13	5.61	0.17	3.6
63–5,0 (enriched with inertinite)				
Initial state	2.11	4.32	0.26	5.6
After exposure to a magnetic field	2.27	4.43	0.37	8.4
< 5,0 (enriched with inertinite)				
Initial state	2.70	3.86	0.46	12
After exposure to a magnetic field	2.79	3.88	0.55	15.3

It should be noted that the change in the value of the parameter concentration of paramagnetic centers (N) is comparable to the measurement error. Therefore, the data presented in Table 5 should be used only for a qualitative assessment of trends in transformations. The EPR spectrum of coal is a superposition of signals of two types of PMC: narrow, belonging to conjugate systems, and wide, associated with the paramagnetism of free radicals. Indicators of the concentration of paramagnetic centers (*N*) in the structure of the substance and the width of the signal (Δ*H*) after exposure to a magnetic field show a slight tendency to increase, which may indicate a slight activation of the substance and the state of the interface systems. The properties of the latter, in particular, include sorption capacity.

The conjugation coefficient (*K*_*sc*_) is calculated as the ratio of the paramagnetic centers associated with conjugate systems to the total number of PMCs in the sample. In the class of coarseness of particles of coal enriched with vitrinite (0.16–0.10 mm), this figure decreases after external influence. It can be assumed that in the molecular structure of vitrinite, there is a redistribution of hydrogen atoms, that is, the conjugate systems of vitrinite are hydrogenated. But at the same time, the molecular structure of inertinite (0.063– < 0.05 mm and < 0.05 mm) is enriched with conjugation systems, that is, dehydrated. The obtained results are in good agreement with theoretical ideas about the redistribution of hydrogen between aromatic and aliphatic components in the process of destructive free radical reactions. The indicator of the threshold sorption capacity (*Q*) is also calculated on the basis of experimental data. The values of the indicator are underestimated (for grade "G") but the tendencies of its change after exposure to a magnetic field are well consistent with the existing ideas about the sorption properties of coal macerals. The sorption capacity of micro components of the inertinite group is growing due to the development of aromatic cluster conjugation systems. The sorption capacity of the macerals of the vitrinite group decreases due to the destruction of the interface systems responsible for interfacial interaction.

Thus, a certain consistency in the change in structural indicators allows us to argue about the influence of weak electromagnetic pulses on the state and properties of the carbon substance. However, the experiment showed that under laboratory conditions, one weak electromagnetic effect is not enough to undergo structural transformations in an amount sufficient to significantly change the properties. External electromagnetic pulses reduce the energy barrier of reactions, which, in natural conditions, is overcome mainly due to the reserve of additional energy accumulated in the microstructure of matter^89^.

The main results of the study of samples of coal of fat fractions of 63–5.0 μm; < 5.0 μm; 160–100 μm; 80–63 μm using FTIR spectroscopy (Nicolet iS10 spectrometer) are as follows. On vitrinite (160–100 μm), the spectrum changes are more significant than those on inertinite (63– < 5.0 μm). At the same time, treatment with an electromagnetic field led to opposite effects on different macerals. The intensity of oscillations of interatomic bonds in inertinite has increased statistically significantly. The difference between the integral areas of the spectra before and after treatment with an electromagnetic field for the class of grain coarseness of < 5.0 μm is 53.339 a.u., for 63− < 5.0 μm is 73.317 a.u.

The opposite result was obtained after processing the grains of coals enriched with vitrinite, there is a decrease in the intensity of oscillations. The difference between the integral areas of the spectra before and after treatment with an electromagnetic field for the grain size class of 160–100 μm decreased by − 97.858 a.u., and, for grains of 80–63 μm, decreased by − 9.922 a.u. One of the possible explanations for this fact may be the destruction of matter due to processes caused in the molecular structure by the effect of a magnetic field.

The peak of 1700–1730 cm^−1^ fluctuations of carbonyl groups increased significantly on a substance enriched with micro components of the inertinite group, especially on the grain size class of 63–5.0 μm. Additional carbonyl groups in the treated substance appeared due to the oxidation of double bonds by oxygen in the air under external influence. A less powerful increase in the intensity of oscillations for the < 0.05 coarseness class is due to the increased content of mineral impurities in the sample. This assumption is confirmed by the presence in the spectrum of coal of a class of coarseness of < 5.0 μm of a non-indicative peak of 1100–1040 cm^-1^ associated with fluctuations in ash components that did not respond to external influences. Increased content of the mineral component of coal is also observed in the grain size class of 200–160 μm. So, peaks in the ranges of 3600–3700 cm^−1^ and 1120–1080 cm^−1^ are associated with silicon compounds and are not indicative in these studies, however, as well as a wide peak of diffuse reflection of 2500–3700 cm^−1^ associated with valence oscillations of water hydroxyls.

The results of laboratory studies by EPR and IRS methods suggest that changes in structural characteristics recorded in the process of experiments with low-energy influences can with high probability be precursors to the transformation of matter or changes in the state of coal in preparation for structural and functional transformations. For example, to sorption interaction or to destructive processes with methane generation. Structural studies have shown that weak magnetic fields affect the structure and properties of metastable coal matter but this influence in the laboratory is not enough to fully repeat natural transformations.

The action of a weak magnetic field initiates spin-selective chemical reactions characteristic of gas and liquid media in coals^85,90^. After treatment with a weak electric field, Table 6, the size of the microparticles of coal decreased by 15–29%.

**Table 6:** Main results of calorimetric analysis of fat coal.

No	Type of processing of a coal sample	Sample mass, mg	Coal particle sizes according to laser diffraction analysis data, μm		Heat of combustion of coal, kJ/kg	Loss of coal mass during heating to 120 °C	
			max	min		mg	%
1	Original	6.24	214.7	116.2	33,564	0.46	7.37
2	Treated with an electric field	11.30	184 decrease by 15%	85 decrease by 29%	33,270	0.24	2.12
3	Treated with a magnetic field	7.88	450 increase by 210%	128 increase by 11%	34,180	0.12	1.52

After treatment with a weak magnetic field, the size of the microparticles increased by 210% (maximum) and by 11% (minimum). The most thermally stable are coals treated with a magnetic field (mass loss is 1.52%). This is evidenced by the results of differential scanning calorimetry, diffractometry of powders, distribution of microparticles by size, etc. The value of coal combustion after treatment with electric and magnetic fields changed slightly. The influence of a magnetic field of weak intensity stimulates chemical reactions aimed at the transition of the system as a whole to a more stable state, characterized by a smaller supply of excess energy.

**Figure 8:**
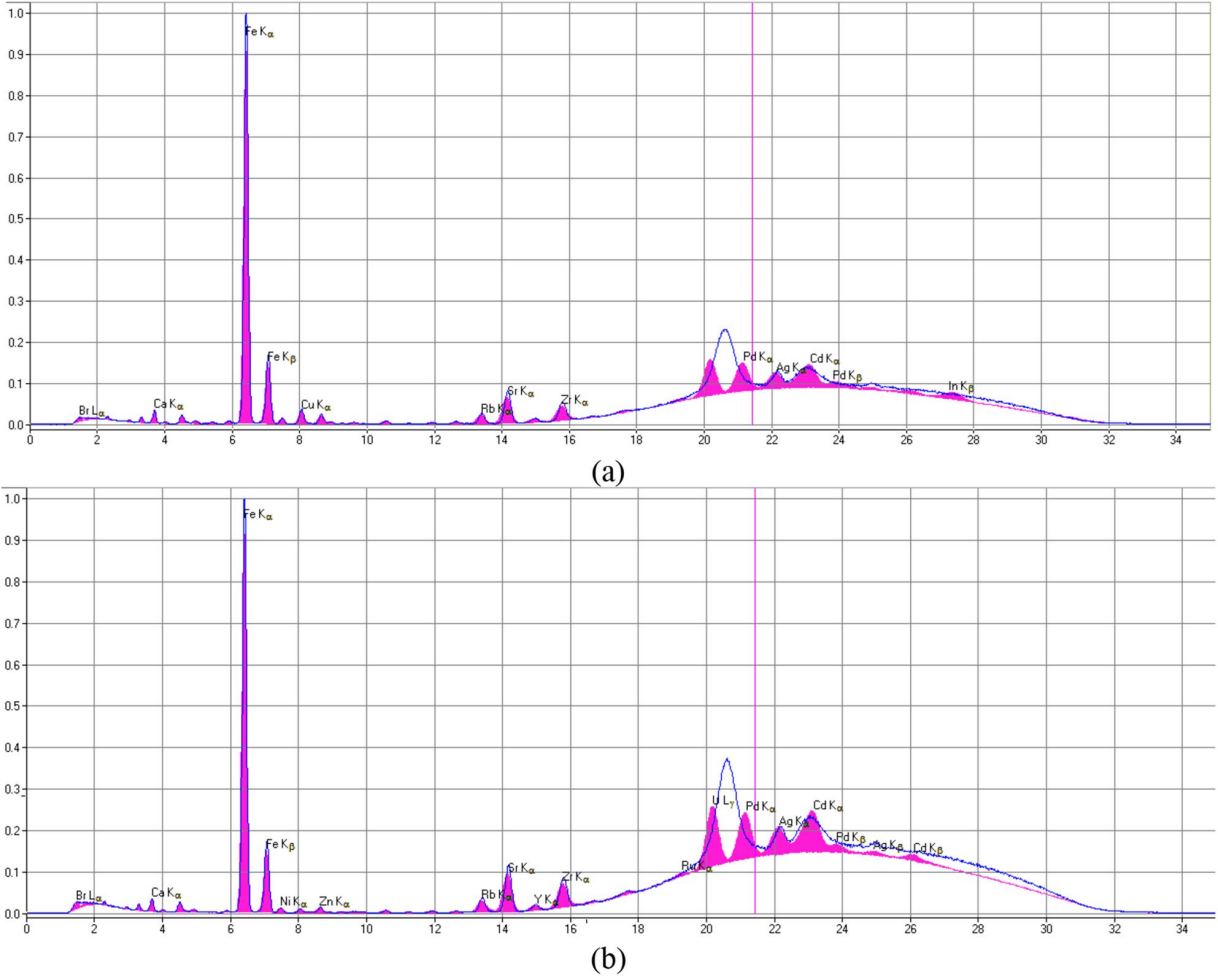
Coal X-ray fluorescence spectra of 53–40 µm fraction (**a**) and 200–160 µm fraction (**b**)^93^.

The results of X-ray fluorescence analysis (Fig. 8), Mossbauer spectroscopy, and nuclear gamma resonance of coals selected from calm zones indicate that the methane content of coals correlates with the total amount of iron^91^, and the outburst hazard of coal correlates only with the presence of ferrous iron^92^. In particular, the following coals of the Donetsk basin were studied using Mossbauer spectroscopy^93^: lean-sintering (LS), selected from the bed *L*_4_ of the «Yasinovka Glubokaya» mine, coke (C) of the bed l_1_ from the 13-bis mine, lean (L), taken from the lava No. 3 of the «Glubokaya» mine. These coals are characterized by a very low intensity of the component associated with ferrous iron; the main amount of iron is contained in pyrite. The spectrum from the coal of the LS grade of the bed *h*_10_ from the Yuzhnaya mine indicates a large amount of iron, which is consistent with the methane content of this reservoir. In the spectrum of this sample, components from ferrous iron also appeared in terms of parameters close to FeSO_4_ × 7H_2_O (the rest of the iron in the form of pyrite FeS_2_). The spectra of lean coal from the 14th lava of the Glubokaya mine, anthracite (A) of the 2–2 bis mine, and anthracite from the Kommunist mine contain only a component of ferrous iron. These coals are outburst hazardous and methane-bearing. The results of the research reported in Ref.^94^ confirmed the pattern previously established in Ref.^93^, and showed that after outbursts in the coals of the Pechersk and Donets basins, the iron content exceeds almost twice its content in non-outburst coals of the same beds.

Paper^93^ reports that "… trivalent iron is concentrated only in flat secretions in the interlayer space of coal, and ferrous iron in spherical clusters, which are more or less evenly distributed in the volume of coal matter". If we assume that nanoscale particles of iron oxides are distributed in the volume of coal matter, then the result of their reaction in an external electromagnetic field may be ambiguous. Nanoparticles stronger than massive material exhibit high values of magnetization, magnetic anisotropy, exchange interaction and abnormally large magnetocaloric effect^95^. These facts require a special procedure for studying the physicochemical interactions of nanoscale iron oxides with coal matter.

Analysis of the X-ray^94^ and Mössbauer spectra^92^ of non-outburst-prone coals indicates a low intensity of the component associated with divalent iron, which may indicate a very low pyrite content. In outburst-hazardous coals, the pyrite content reaches 20%. After the outburst of coal and gas, the amount of Fe^3+^ ions increases while the Fe^2+^ ions are redistributed in crystal hydrates (40%), siderite (~ 12%), silicates with Fe^2+^, sulfates of the type FeSO_4_ × *n*H_2_O. In addition to pyrite, hematite and X-ray amorphous oxygen-containing forms of iron are catalytically active as "external" catalysts^96^.

In^97^, using the example of the Fe–O–C–CO_2_–H–CH_4_ system, it was shown that the transformation Fe_2_O_3_ → Fe_3_O_4_ is thermodynamically possible, and in the presence of carbon or carbon dioxide, the beginning of the transformation occurs at a temperature of 338 K. Transformations can also occur at temperatures below 338 K due to a decrease in the reaction barrier. There is a chemical relaxation of the additional energy reserve that was stored in the microstructure of coal during the period of tectonic activity. The influence of the electric field leads to the formation of a phase with higher electrical conductivity, as shown in Ref.^98^.

There is no still reasoned answer to the question of the reasons for the formation of a high content of ferrous iron in outburst-hazardous coals and an additional amount of methane in connection with the possible catalytic effect of iron has not yet been answered. It is possible that the processes of formation of methane and ferrous iron are independent of each other and parallel, activated when the temperature of the carbon substance increases. With simultaneous exposure to the electric field system, the threshold of chemical reactions may decrease. However, in all cases, the criterion for the outburst hazard of coal remains a high content of ferrous iron.

Probably, at certain temperatures, and concentrations of mobile components, the reduction of iron will be influenced by the process of thermal oxidation. On the example of experimental studies of siderite dissociation under heating and simultaneous electromagnetic influence, the peak temperature of the reaction decreases by 130–250 K^57,98^.

X-ray fluorescence spectroscopy is used in the study of samples of reduced gas coal after treatment with electric and magnetic fields. Coal samples were taken from bed *K*_8_ of the Dimitrov mine (Myrnograd, Ukraine). As is known, the reduced coal is characterized by a high iron content^95^. The ash content of coal is Ad = 10.7%. From 1.0 kg of coal, 107 g of ash was obtained, which contains 2.85% iron, 54% quartz, and other compounds.

**Figure 9:**
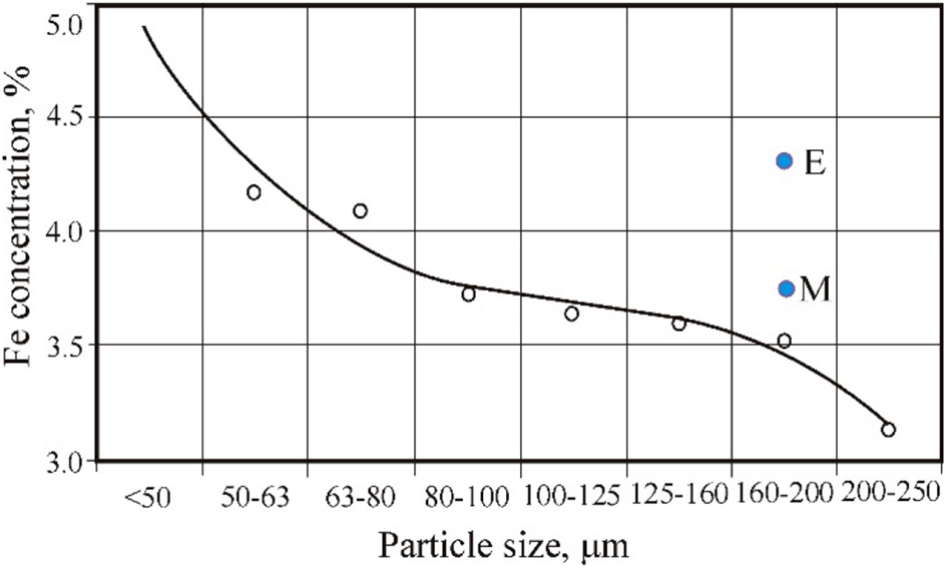
The iron concentration changes in the reduced gas coal depending on the fractional composition: Points M and E are the coal of 200–160 µm fraction treated by the magnetic and electric field of low intensity^93^.

The energy value for the lines *K*_*α*_ and *K*_*β*_—Fe is 6.4 keV and 7.02 keV, respectively. In the energy range from 16 to 32 keV, the area under the curve reflects the background created by the device. Analyzing the spectra of fat coals from outburst non-hazardous zones and coal after an outburst, obtained in Ref.^78^ was established, that under the curve the background area grows with a decrease in the contribution from iron. A similar picture is observed when studying samples of gas coal in the range of grains from 40 to 250 μm. The curve in Fig. 9 reflects a trend that indicates an increase in iron concentration with a decrease in the size of microparticles (gas coal), i.e., practically repeating the results of similar research with fat coal^78^. It is known that with a decrease in particle size, the chemical activity of coal increases^28^.

After magnetic treatment, the concentration of iron increased to 3.72% relative to untreated (3.53%) coal. For comparison, the value of the concentration of iron after exposure to an electric field (due to the grinding of microparticles) is shown—4.34% of iron. On the surfaces of phase transitions, destructive processes occur. Part of the stationary components of the condensed phase, collapsing, passed into mobile. It should be noted that in coal beds, one of the main signs of outburst hazard is the "grinding" (fine grinding) of coal.

It follows from the data of X-ray fluorescence spectroscopy of coal powders of various fractions (from 250 μm to < 50 μm) that with a decrease in the fraction, the concentration of iron increases. In general, an increase in iron with a decrease in the size of microparticles can be associated with an increase in the number of exposed inclusions on the newly formed surfaces—condensed phases containing iron. One of the indicators of coal after an outburst is an increase in iron concentration^98^ and a decrease in particle size^83^, an electret state^64^ and other indicators^6,10^. Coal after exposure to an electric field acquires the same characteristics. Accordingly, there is reason to assert that electromagnetic factors play a certain role (among all others) in the formation of outburst-hazardous coals.

An unexpected result of the simultaneous effect of a weak electric field when heating samples of siderite (dielectric) was that a new phase was formed in the intergranular boundaries of siderite by a jump, possessing an electronic type of conductivity^57^. The electrically conductive phase in siderite is not formed by the action of only temperature or only an electric field. In coal research, this previously unknown physical effect is interesting from the point of view of the mechanism of formation of electrically conductive phases with an electronic type of conductivity, in particular, carbon phases (graphene, graphite, etc.) and iron oxides.

Breaking chemical bonds is energetically more favorable for electroneutral decay products than for ions^89^. For the chemical bond of two atoms A–B, the decay variants have two possible paths:

3 $$A{-}B \, \to \, A + B \,\,\,\,\,\,\,\,\,\,\,\,\,1.U_{A - B} ,$$

4 $${\text{A}}{-}{\text{B }} \to {\text{ A}}^{ + } + {\text{B}}^{ - } \,\,\,\,\,\,\,\,\,\,{2}.{\text{ U}}_{{{\text{A}} - {\text{B}}}} + \, \left( {{\text{J}}_{{\text{A}}} {-}{\text{A}}_{{\text{B}}} } \right).$$

In the first case, the energy input is equal to the strength of the bond U_A-B_, and in the second case, it is necessary to bring an energy equal to the difference in the ionization potential J_A,_ and the means to electron A_B_. For organic compounds, the agent to the electron of radicals can reach 2 eV. However, the ionization potential of their fragments, as a rule, is not lower than 6 eV. Therefore, the decay of organic compounds occurs mainly along a radical or molecular path^89^. Along with thermal activation, the additional effect of the electric field stimulates phase transformations in the system aimed at increasing electrical conductivity^56,91^.

On the diffractograms of coal, after treatment with weak magnetic and electric fields (Fig. 4), such crystalline substances were identified by characteristic reflection maxima d/n = 4647 as quartz (3659; 334; 2697; 2449), pyrite (3,12; 2,96; 2,408; 2,21; 2,11). Chemical analysis of the ash showed that more than half of the mass belongs to α quartz. Diffraction maxima in the range of interplane distances d/n = 8.56…6.51 and 3.8…3.56 correspond to kaolin, and the maxima in the range of interplane distances d/n = 2.78; 1.74; 1.498; and 1.01 belong to siderite.

The diffraction pattern of gas coal contains a wide maximum at an angle of 2θ ~ 27°40′. The maximum radiation intensity (for SiO_2_, the line is 0.334 nm) for the original sample is 94%, for the one treated with a magnetic field—110%, for the one treated with an electric field—42%. Analysis of diffractograms and comparison of the results obtained with data from studies of changes in the fractional composition of coal indicate a change in the size of microparticles. At the angle of 2θ ~ 27°40′ there is a maximum, the half-width of which reflects the degree of ordering of the structure. The ratio of maxima of the relative intensities at an angle of 2θ ~ 27°40′ for coal treated with an electric field and coal after magnetic field treatment is ~ 1:2. After magnetic treatment, the degree of the orderliness of the coal structure increased significantly compared to the original and relative to the coal treated with an electric field.

**Figure 10:**
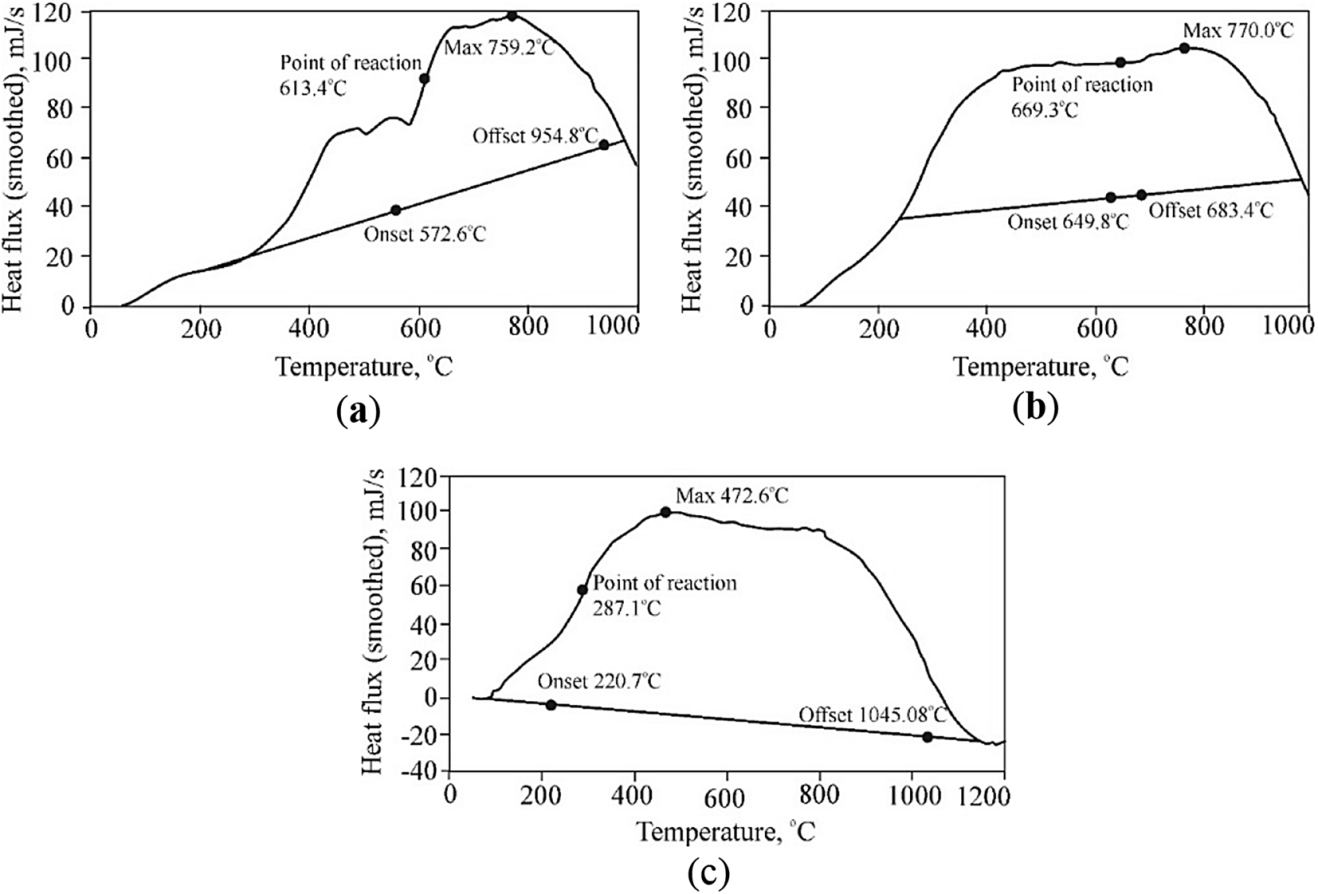
Results of differential thermal analysis of 200–160 µm gas coal fraction: (**a**) untreated coal (pieces); (**b**) coal treated by a magnetic field; (**c**) coal treated by an electric field.

Determination of phase transitions and thermal effects in samples under changes in temperature and mass of coal, determination of enthalpy, the temperature of endothermic and exothermic processes, Fig. 10, were performed using thermogravimetry, differential scanning calorimetry, and differential thermal analysis using a Netzsch STA 449 F3 Jupiter instrument. For the convenience of analyzing the results obtained, the main indicators for each experiment are given in Table 7.

**Table 7:** Main indicators of differential thermal analysis of gas coal of fractions 200–160 μm.

No	Sample of gas coal from bed K_8_ in dimitrov mine (reduced coal)	Temperature of chemical reaction, °C		Enthalpy of new phase formation, kJ/kg	Reaction point *T*, °C	Max *T*, °C	Heat flux mJ/s
		onset	end				
1	Original coal (not crushed, pieces)	572.6	954.8	+ 2484.725	613.4	759.2	117.11
2	Crushed coal (original 200/160)	258	565.9	+ 4991.564	269.8	488.1	98.979
3	Treatment of crushed coal with a magnetic field	649.8	683.4	+ 3279.787	669.3	770.0	107.18
4	Electric field treatment of crushed coal	220.7	1045.8	+ 5777.176	287.1	472.6	100.26

In chemical reactions occurring in coals at atmospheric pressure, the measure of the isobaric thermal effect of the reactions is the change in enthalpy, which is a positive value. This result suggests that the formation of new chemical compounds occurs with the absorption of heat.

The greatest positive value of enthalpy corresponds to the treatment of coal with an electric field, the action of which leads to the destruction of the weakest bonds of mainly carbon and hydrocarbon chains, the destruction of the surface atomic layers of coal microparticles, the formation of new gas molecules. The enthalpy of coal treated with an electric field exceeds the value of the enthalpy of the samples after exposure to a magnetic field by 2.5 MJ/kg. In both cases, heat is absorbed by the system to form new compounds, but in the case of the influence of a magnetic field, some of the chemical reactions pass through the mechanism of spin polarization. In addition to changes in enthalpy, the value of the heat of combustion decreases: the original coal (crushed)—33,554 kJ/kg; after treatment with a magnetic field—34,180 kJ/kg; after treatment with an electric field—33,070 kJ/kg. A similar trend is observed in the processing of fat coal (Table 7). This effect may indicate an increase in the ratio of C/H, i.e., a significant "loss" of hydrogen associated with the formation of new hydrocarbons (methane, acetylene, and other gases, including heavy hydrocarbons).

On Fig. 10 and Table 7 can be seen that the temperature range of chemical reactions is highly dependent on the effects of weak magnetic and electric fields (in comparison with the coals of the original samples). This is evidenced by the values of enthalpy, the heat of combustion, the size of microparticles, X-ray diffraction analyses, the values of sorption and desorption of methane from gas-saturated coal samples, the value of electret potentials of samples of outburst-hazardous coals and those treated with weak electromagnetic fields. The logic of the results includes the data obtained in the study of spectra obtained by the methods of IR, CS, EPR, NGR, ^13^C NMR, and ^1^H NMR spectroscopy.

The effect of physicochemical transformations under the influence of weak magnetic and electric fields is manifested to the maximum extent in coals of the initial stage, decreasing with increasing degree of coalification. The fundamental difference in the development of chemical reactions in coals when treated with magnetic and electric fields, is as follows. The mechanism of influence of a weak magnetic field is a magnetically stimulated effect on particles and does not depend on temperature, and the effectiveness of the electric field depends on temperature and increases with its growth.

For the first time, experimental results of complex physicochemical research of hard coals with a pre-destabilized microstructure, treated with weak electric and magnetic fields, have been obtained. A comparative analysis of the properties of coals selected from non-outburst-prone areas of the bed, after their grinding and electrical activation, with coals of natural outburst hazard indicates the identity of their properties. In general, the difference between the electric activation method and the well-known mechanical one is the initiation of new physicochemical transformations in the nanostructure of coal. In particular, this method expanded the understanding of magnetic spin phenomena associated with the behavior of the spin of electrons and nuclei in solid-phase chemical reactions, the catalytic function of Coulomb centers, the role of crystal structure defects in activating the formation of new carbon and other phases.

Prospects for further research and practical use of the effect of weak electromagnetic fields are associated with suppressing or reducing the potential for outburst hazards through magnetically selective stabilization of the coal microstructure, the application of an electric field to activate chemical reactions in coal hydrogenation technologies, and coal mine methane production. An important direction in the development of the results obtained is the development of practically significant methods for controlling the state of the carbon rock mass, that is, control over structural transformations in the substance, subject to an adequate assessment of its physicochemical state. Such physical and chemical transformations are natural and develop due to the energy release accumulated in the microstructure of a substance with additional excitation of the macrosystem by mechanical, thermal, or weak electromagnetic fields.

The ground for such statements is the fact that electric fields stimulate chemical reactions leading to structural transformations in the substance of coals, and the role of the magnetic field is to initiate spin transitions with the subsequent transformation of the molecular structure of coals into a more energy-efficient configuration.

## Conclusions

The authors presented new experimental results in the research on the effects of weak electric and magnetic fields on hard coal samples. When comparing the actions of electric and magnetic fields on coal patterns from the same sample, in some experiments the results differ by only a few percent, but in most experiments the difference is fundamental.

After treatment with a weak electric field, coal acquires electret potential as a result of passing the weak electric current with a density of 10^–6^ – 10^–5^ A/mm^2^ (coal temperature up to 320 K, duration of current passage is 4 h). The relaxation time of the electret charge exceeds the Maxwellian time (30 s) by more than four orders of magnitude. Outburst-prone coals are characterized by similar properties. Gas desorption from methane-saturated coal treated with an electric field, in comparison with the original coal, decreases by only 3–5%, but at the same time, the rate of methane emission increases significantly: 4.2 times for long-flame coal, 3 times for gas coal, 1.7 times for fat coal and 1.24 times for coking coal. As coal approaches anthracite (or as carbon content increases), the gas saturation of coal samples decreases. Electrical stimulation of coal leads to a decrease in the size of microparticles in the studied range of 214–162 microns. The action of an electric field on particles in the range of 214–96 microns increases the yield of the smallest microparticles by reducing the yield and size of large ones.

The enthalpy values in the formation of a new phase decreases; overall chemical activity increases. Compared to the original coal, the maximum gas outlet temperature is significantly reduced. The mass loss decreases by 3.5 times when coal is heated to 397 K, which is probably due to the formation of mobile components and their release during electrical stimulation. The iron concentration after electric field exposure (due to grinding of microparticles) is 4.34%, which is 23% higher than the iron concentration in the original coal sample. The characteristics of the crushed coal after electric field exposure differed little from the characteristics of the coal after the outburst. It is assumed that in the formation of outburst-prone coals, a certain contribution (in addition to all others) is made by the electromagnetic factor.

In some experiments on magnetic stimulation of chemical reactions, the results obtained differ noticeably or fundamentally from the results of electrical stimulation. The size of microparticles fractions 200/100 μm increases (Table 6) by 210 and 11% due to magnetically stimulated solid-phase reactions, and the heat of combustion—by 2%. The weight loss decreases by 5 times when heated up to 397 K. The temperature range of chemical reactions after magnetic treatment was (650–683 °C) of the original sample and the one treated with an electric field, respectively (573–955 °C) and (221–1046 °C), which indicates greater resistance of the microstructure to thermal activation of chemical reactions. After magnetic treatment for particles of grain size 200/160, the iron concentration increased to 3.72% relative to particles of the same size range, but untreated (3.53%) coal.

## Data Availability

All data generated or analysed during this study are included in this published article.
